# Efficacy testing of the SAVOR (Sisters Adding Fruits and Vegetables for Optimal Results) intervention among African American women: A randomized controlled trial

**DOI:** 10.34172/hpp.2020.41

**Published:** 2020-07-12

**Authors:** LaVonne Brown, Manoj Sharma, Sophia Leggett, Jung Hye Sung, Russell L. Bennett, Mario Azevedo

**Affiliations:** Behavioral & Environmental Health, School of Public Health, Jackson State University, MS, USA

**Keywords:** Fruits, Vegetables, Behavior, Program evaluation

## Abstract

**Background:** In the United States, only about 15% of individuals meet daily fruit intake recommendations of 2 cups per day and only 10% meet the vegetable intake recommendations of3 cups per day. African American women are a high-risk group. In this study, a fourth-generation multi-theory model (MTM) of health behavior change was used to design and evaluate a SistersAdding Fruits and Vegetables for Optimal Results (SAVOR) intervention for AA women.

**Methods:** The study utilized a randomized controlled trial (RCT) with measurements taken at pretest, posttest (after the three-week intervention) and follow-up (at the end of eight weeks).SAVOR (n=26) was compared to an equivalent knowledge-based intervention (n=28). Process evaluation was done for program fidelity and satisfaction. A validated 38-item self-reported questionnaire was used to measure changes in MTM constructs and past 24-hour consumption of fruits and vegetables.

**Results:** The SAVOR intervention resulted in improvement of mean consumption of fruits and vegetables in the experimental group from pre-test (2.78) to posttest (4.77) to recommended levels at follow-up (5.04) while in the comparison group they remained at around 3 (P<0.0001)Statistically significant changes (P<0.05) were noted for all MTM constructs except for participatory dialogue.

**Conclusion:** The SAVOR intervention was found to be efficacious and established the robustness of MTM. SAVOR can be replicated for future effectiveness trials.

## Introduction

Globally, 1.7 million deaths (2.8%) are attributed to not consuming adequate amounts of healthy balanced diets rich in fruits and vegetables.^1^The Global Burden of Disease (GBD) identified diets low in fruits and vegetables as one of the five leading risk factors contributing to chronic diseases around the world.^2^ Fruits and vegetables are a rich source of vitamins, minerals, dietary fiber, and dietary bioactive content that are associated with reduced risk for coronary heart disease, cancer, hyperlipidemia, cognitive impairment, macular degeneration and other chronic diseases.^3^ United States Department of Agriculture (USDA),in 2015-2020 Dietary Guidelines for Americans, recommends consumption of 2 cups of fruits and 3 cups of vegetables per day for adults. In the United States, only about 15% of individuals meet daily fruit intake recommendations and only 10% meet the vegetable intake recommendations.^4,5^

African Americans when compared to Caucasian Americans experience higher prevalence and mortality rates related to heart disease,^6^ stroke,^7^ diabetes,^8^ cancer^9^ and other chronic diseases in which fruits and vegetables play an important protective role. Obesity rates are also higher among African Americans, especially women when compared to White counterparts.^10^ Accompanying these disparities in disease conditions and risk factors there is a well-established disparity in availability and consumption of fruits and vegetables among African Americans compared to Whites that leads to decreased intake among African Americans.^11,12^ Thus African Americans constitute a high-risk group.

Some interventions with the African American community have been designed to promote fruits and vegetable consumption. A pilot beauty salon-based intervention in South Carolina, *Steps for a New You* , with African American women was able to demonstrate an increase in mean consumption of fruits and vegetables in the experimental group compared to control group despite a very small sample size.^13^ Another intervention has been described called *Healthy Eating and Harambee* (meaning “all pull together” in Swahili) was based on PEN-3 model and trans-theoretical model.^14^ A different North Carolina church-based intervention for African Americans included the components of peer counseling, pastoral advisement, and other educational and environmental changes; however, it was not able to demonstrate any statistically significant changes in fruits and vegetable consumption.^15^Scarinci and colleagues tested a community-based, randomized clustered intervention in Alabama with African American women. Findings showed an increase in fruit and vegetable intake at a 12-month follow-up. The theoretical framework utilized in this study were Bandura’s social cognitive theory (self-efficacy) and Bandura’s trans-theoretical model.^16^ While a similar pilot intervention delivered in Louisiana at a local community center among African American adults showed ineffective findings.^17^ Barnidge and colleagues utilized the previously existing community gardens in the African American neighborhoods. Frameworks utilized to guide this study were social cognitive theory and community mobilization model. Published mid-intervention results indicated a favorable trend toward perceived fruit and vegetable consumption.^18^ Springfield and colleagues delivered the intervention, *Doing Me! Sisters Standing Together for a Healthy Mind and Body* , as of yet the results of its evaluation have not yet been published.^19^ Brewer and colleagues tested a comprehensive community-based cardiovascular disease prevention program on a consenting group of African Americans in the nation’s Midwest region; the study was entitled, “*Fostering African-American Improvement in Total Health (FAITH!)* ” that showed an efficacious outcome. Frameworks utilized in this intervention were based on the health belief model, community mobilization models, and the social cognitive theory.^20^ Finally, Ansari and colleagues, *Health is Power (HIP)* , examined the role of religiosity in improving fruit and vegetable consumption among minority women including African Americans. The intervention utilized multiple theories ecological theory, social cognitive theory, group cohesion models, and decisional balance from the trans-theoretical model. This intervention showed an increase in the health behavior of fruit and vegetable consumption by promoting a theologian component.^21^

Based on this review of existing interventions, the interventions that did not include a theoretical component (evidence-based) were found to be ineffective and unsuccessful.^15-17^ It is apparent that interventions that have used multiple theories are generally successful.^13,16,20,21^ However, the use of multiple theories has been haphazard. There is a need to systematically integrate multiple theories to enhance the effectiveness of behavioral interventions.^22^ One such fourth-generation multi-theory precision approach is imbibed in the multi-theory model (MTM) of health behavior change.^23,24^ Thus, the purpose of this study was dual: 1) to promote fruits and vegetable intake among a group of faith-based African American women; and 2) design, implement and evaluate a fourth-generation intervention based on MTM of health behavior change. The intervention was called SAVOR (Sisters Adding Fruits and Vegetables for Optimal Results). Such trials are called Phase IIb trials according to the Obesity-Related Behavioral Intervention Trials (ORBIT) model.^25^

MTM has been found to successfully explain physical activity behavior change in college students, portion size consumption behavior change in young adults, sleep behavior change in college students, changing consumption of sugar-sweetened beverages to water among youth, physical activity behavior change in African American women, changing binge drinking behavior to responsible drinking behavior among college students, and low salt intake behavior change in hypertensives.^26-32^ In interventional studies, MTM has also been found to be efficacious for promoting physical activity behavior change and water-pipe smoking reduction.^33-34^ MTM has been applied to predicting fruit and vegetable consumption in a sample of college students and African American women.^35,36^

## Materials and Methods

### Design

**Figure 1:**
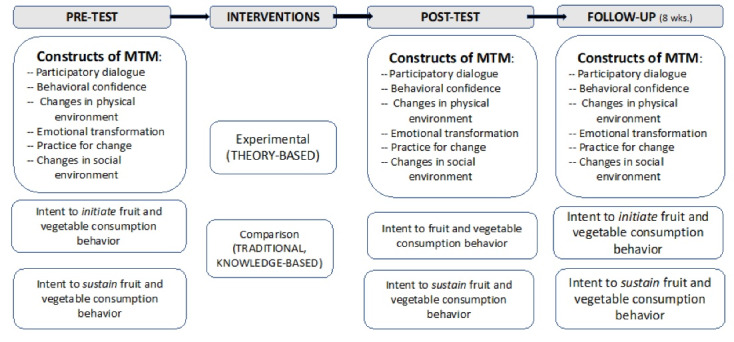
Fruit and vegetable intervention logic design.

The study used a randomized controlled trial (RCT) with measurements taken at pretest, posttest (after the three-week intervention) and follow-up (at the end of eight weeks). Rationale for measurement time points for this study were based on allowing enough time for participants to begin the targeted behavior and continue a pattern beyond the intervention. The experimental arm received the MTM-based three-week intervention while the comparison arm received an equivalent knowledge-based intervention delivered didactically. A knowledge-based intervention was chosen due its traditional and practical method to deliver pathology of fruit and vegetable consumption and related data associated with its effects on the human body. RCTs are considered the gold standard for efficacy testing and the process of random assignment distributes all potentially confounding variables equally between the groups. The between-subjects independent variable was the group with two levels (experimental and comparison), the within-subjects independent variable was time with three levels (pretest, posttest, and follow-up), and the dependent variables were the means scores on the constructs of MTM, the means scores on the intentions to initiate and sustain behavior change with regard to fruits and vegetable consumption, and mean of total number of fruits and vegetables consumed during the past 24 hours. The study design is depicted in Figure 1.

### Population and sample

The setting for the study was in the Greater Jackson area of Mississippi. According to the US Census Bureau (2018) there were 88 491 African American women in this area. For this study, women who self-identified themselves as African American or Black; were 18 years of age or older residing in Greater Jackson area of Mississippi; and not currently consuming the recommended servings of fruits and vegetables (total of five cups per day) were recruited from four local churches representing both Baptist and Church of Christ denominations. For sample size estimation, a power analysis was conducted for repeated measures analysis of variance (ANOVA) using G*Power.^37^ An alpha of 0.05, a power (1-beta) of 0.80, assumed effect size f of 0.30 (medium) (as is the case in social and behavioral science research), number of groups being 2, number of measures being 3, correlation among repeated measures as 0.5 and non-sphericity epsilon as 1 yielded a sample size of 20 in each group.^38^ This was inflated by 50% to account for any attrition or missing values. So, the target recruitment in each group was 30 women.

### Instrumentation

A 38-item self-report instrument was used for this study that has two items about fruit and vegetable consumption behavior, seven demographic questions, five items pertaining to advantages, five items pertaining to disadvantages, five items pertaining to behavioral confidence and three items each for changes in the physical environment, emotional transformation, practice for change, and changes in the social environment, and one item each for intention to initiate and sustain behavior change. The instrument has been validated for face validity, content validity, construct validity, and internal consistency reliability with African American women in a prior study.^36^

### Interventions

**Figure 2:**
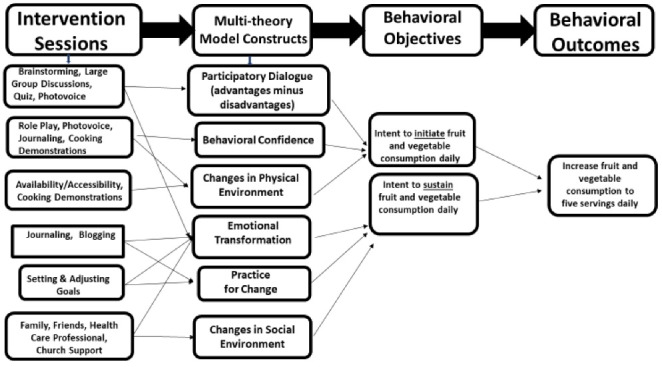
Logic Design depicting SAVOR intervention sessions, MTM constructs, behavior objectives, and behavioral outcomes

Both the experimental SAVOR and comparison interventions were three weeks long with three sessions meeting weekly for 60 minutes. The SAVOR intervention was delivered by a Certified Health Education Specialist (CHES) with guest speakers from the Mississippi Urban League/Mississippi Roadmaps to Health Equity and The Food Factor, Mississippi State Extension Service. The initiation construct of the *participatory dialogue* was fostered through brainstorming, large group discussion, quiz, photovoice; *behavioral confidence* was developed through role play, photovoice, and cooking demonstrations; and the construct of *changes in the physical environment* was developed through providing fruits and vegetables to the participants. The construct of *emotional transformation* was developed through psychodrama; *practice for change* was built through social media and journaling; and the construct of *changes in the social environment* was developed through social support. The linkages between activities and MTM constructs are depicted in Figure 2. Participants were asked to practice journaling by record eating behaviors and patterns (what fruits and vegetables were eaten at home or away from home, how often they ate) document their physical and emotional eating challenges and successes. Weekly text messages were sent electronically to participants in the SAVOR intervention between intervals of the post-test and eight-week follow-up. A sample kit of fruits and vegetables, one high fiber fruit bar, one measuring cup, a storage container, one journal, one rubber spatula, and numerous health related pamphlets were given to each participant at the completion of the intervention. Make-up sessions were conducted after early worship services in lieu of Sunday school and immediately after Wednesday night bible study services at participating churches.

The comparison intervention was imparted knowledge through lectures and assignments about the daily recommended fruit and vegetable intake per USDA, appropriate serving or portion size, identification of various fruit and vegetables, benefits of fruits and vegetables, disadvantages of consuming insufficient amounts of fruits and vegetables, disease consequences and health conditions associated with lack of fruit and vegetable consumption, theories to promote fruit and vegetable consumption, and importance of consuming a variety of fruits and vegetables as opposed to counting calories.

### Process evaluation

The participants in both groups completed a session rating form to gauge satisfaction with the interventions. The scale ratings were fair, good, very good, and excellent. Participants were asked to evaluate the program based on the following categories: (1) learning from the sessions, (2) content relevance, (3) session activities, (4) content effectiveness, (5) session pace, (6) allocated time, (7) researcher’s facilitation methods, and (8) likelihood of participating in future research. In addition, participants in both groups were observed by two CITI-trained observers who completed checklists of tasks performed in each session and recorded the time spent on each activity (for measuring the degree of program fidelity). All process evaluation tools were validated for face and content validity in a two-round process by a panel of five experts.

### Data analyses

All data were analyzed using SPSS, version 25.0 (Armonk, NY: IBM Corp.). Descriptive statistics for demographic and study variables at pretest, posttest, and follow-up were computed in the form of frequencies and percentages for categorical variables and means and standard deviations for metric variables. Differences between demographic variables and study variables between experimental and comparison groups at pretest were analyzed using the chi-square test for categorical variables and two-tailed F-test for metric variables. Mean differences in scores for study variables between pretest, posttest and follow up for experimental and comparison were compared using repeated-measures ANOVA test. Sphericity assumed within-subject effects tested using the Mauchly’s test were reported. The significance level (α) was set at *P* < 0.05. For the initiation model that tested the intention for starting fruits and vegetable consumption, since participatory dialogue showed significant difference at pretest between experimental and comparison group, repeated measures analysis of covariance (ANCOVA) was applied with it being the covariate. Likewise, for the sustenance model that tested the intention for maintaining fruits and vegetable consumption, since emotional transformation and changes in social environment showed significant difference at pretest between experimental and comparison group, repeated measures ANCOVA was applied with these two being the covariates.

## Results

**Figure 3:**
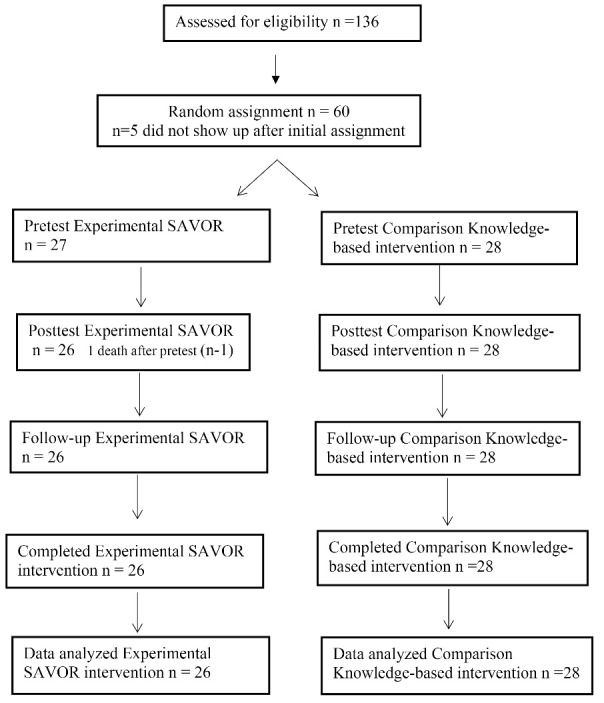
Participants flow through the study and reasons for dropout.

A total of 60 African American women were recruited for this study. Figure 3 summarizes the flow of participants through the trial. For analysis, per-protocol analysis method as opposed to intention-to-treat analysis was used since the loss of participants was very small. Per-protocol analysis is justified in pragmatic trials for comparing the treatment and comparison groups and includes only those patients who completed the treatment originally allocated.^39^

Table 1 summarizes the comparison of demographic variables between the experimental and comparison groups at pretest. It is evident from Table 1 that none of the demographic variables were statistically significant between the experimental and comparison groups. The mean age of the participants in the experimental group was 53.74 (13.94) years while in the comparison group it was 47.93 (19.97) years but the two means were not statistically significant (*P* = 0.22).

Table 2 summarizes the comparison of means and standard deviations and the statistical testing of study variables in the experimental and comparison groups at the pretest. There were no significant differences in the means scores for behavioral confidence, changes in the physical environment, practices for change, intention to sustain fruit and vegetable consumption, fruit consumption, vegetable consumption, and fruit and vegetable consumption together between the experimental and comparison groups. However, participatory dialogue (*P* = 0.003), emotional transformation (*P* = 0.03) and changes in the social environment (*P* = 0.03) were statistically significant between the experimental and comparison groups. In order to account for these differences, repeated measures ANCOVA was performed in initiation and sustenance models for intentions models. Use of ANCOVA did not alter the results from simple repeated measures ANOVA.

**Figure 4:**
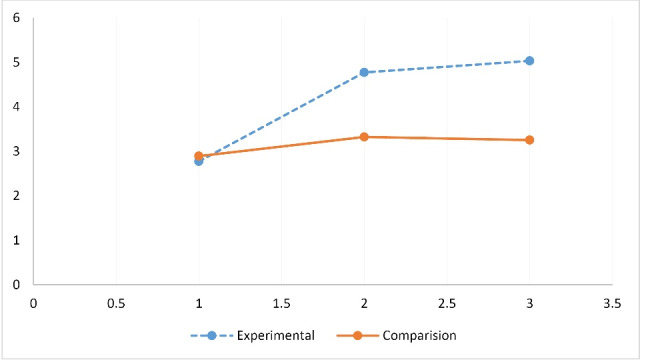
Group x time effect for fruit and vegetable consumption.

Table 3 presents the means and standard deviations of all study variables at pretest, post-test, and 8-week follow-up and the results of repeated measures ANOVA. The mean consumption of fruits and vegetables in the experimental group increased from the pre-test (2.78) to posttest (4.77) and were at the recommended levels at follow-up (5.04) while in the comparison group they were more or less static at pretest as 2.89, at posttest 3.32, and at follow-up 3.25. This is depicted in Figure 4. The effect size of this change as measured by partial eta squared was 0.193.

**Figure 5:**
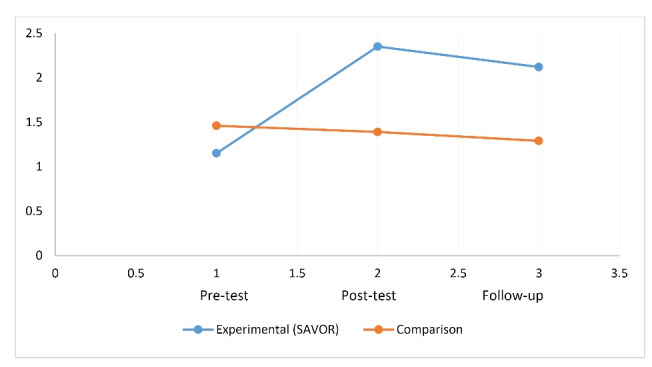
Group x time effect for fruit consumption.

Table 3 also shows that the mean consumption of fruits in the experimental group increased from the pre-test (1.15) to post test (2.35) and were at the recommended levels at follow up (2.12) when compared to comparison group in which the consumption at pretest was 1.46, at posttest 1.39, and at follow-up 1.29. This is depicted in Figure 5. The effect size of this change as measured by partial eta squared was 0.174.

**Figure 6:**
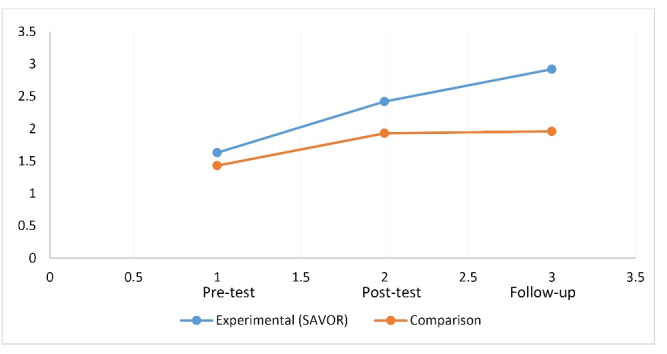
Group x time effect for vegetable consumption.

Table 3 also shows that the mean consumption of vegetables in the experimental group when compared to comparison group increased from the pre-test (1.63) to post test (2.42) to follow up (2.92) while in the comparison group the mean was more or less static at pretest as 1.43, at posttest 1.93, and at follow-up 1.96. This is depicted in Figure 6. The effect size of this change as measured by partial eta squared was 0.072. Likewise, from Table 3 it is evident that all the interaction terms (group x time) for constructs of MTM except participatory dialogue were statistically significant.

### Process evaluation results

The degree of program fidelity was measured by a rater’s completion of a tally sheet for each of the activity in each of the sessions in both the experimental and comparison groups. The results for the percentage of the tallied checked marks indicated 100% adherence to planned activities for both arms. Both the programs were delivered in alignment with the design.

The degree of program satisfaction indicated that the experimental program had statistically greater satisfaction in areas of (1) learning from the sessions (*P* = 0.02), (2) content relevance (*P* = 0.001), (3) session activities (*P* = 0.004), (4) content effectiveness (*P* = 0.002), (5) session pace (*P* = 0.04), (6) allocated time (*P* = 0.012), (7) researcher’s facilitation methods *P* = 0.006). However, all the participants in both the groups indicated their likelihood of participating in future research.

## Discussion

The results of this study showed that MTM was a useful framework in influencing fruit and vegetable consumption among African American women. The mean fruits and vegetables consumption together in the SAVOR group increased from the pre-test level of 2.78 to posttest level of nearly recommended levels of 4.77 to the recommended levels at follow-up of 5.04 while in the comparison group they were more or less static at around 3 (*P* < 0.0001). Corresponding increases were also noted in fruit consumption (*P* < 0.0001) and vegetable consumption (*P* < 0.02) alone. Likewise, all the constructs of MTM except participatory dialogue were statistically significant for the SAVOR group over the comparison group from pretest to posttest to follow-up. The findings lead us to believe that MTM-based SAVOR intervention was efficacious in influencing the outcomes of fruits and vegetables consumption in African American women. These findings further substantiate the findings of a cross-sectional study done with African American women that found that all the three constructs of MTM namely participatory dialogue, behavioral confidence, and changes in the physical environment explained a large portion of variance in the intention for initiating fruits and vegetables in African American women.^36^ However, in this study the interaction term for participatory dialogue was not found to be significant on repeated measures ANOVA. The reason for this could be that women in the SAVOR group of this sample were already convinced of the benefits of consuming fruits and vegetables as evidenced by their high means scores at pretest. However, these differences at pretest were adjusted through repeated measures ANCOVA where participatory dialogue was used as a covariate for explaining the interaction for intention for initiating fruits and vegetables consumption and still the interaction remained significant.

The study by Brown and colleagues also found that the three constructs of emotional transformation, practice for change, and changes in the physical environment accounted for a large proportion of variance in the intention to sustain fruits and vegetable consumption among African American women. These findings were also confirmed by the efficacy trial of SAVOR intervention. However, there were statistically significant pretest differences in means for emotional transformation (*P* = 0.03) and changes in the social environment (*P* = 0.03). These were also adjusted as covariates through repeated measures ANCOVA for explaining the interaction for intention for sustaining fruits and vegetables consumption and still the interaction remained significant.^36^

Previous effective interventions have utilized the construct of self-efficacy and found it to be beneficial.^13,20,21^ The construct of self-efficacy is very much related to the construct of behavioral confidence and this study also supports the usefulness of this aspect in developing efficacious programs for promoting fruits and vegetables among African American women. Just like the SAVOR program demonstrated that this construct can be built through role play, photovoice, and cooking demonstrations, other interventions can also utilize this approach in fostering behavioral confidence.

The construct of changes in physical environment has been used by very few interventions with two interventions showing positive effect while another one showing no effect.^15,17,20^ The SAVOR program underscored the usefulness of this construct which is especially important for low-income minority groups who may not be able to have access to fruits and vegetables because of cost. It is imperative that environmental and policy initiatives be undertaken to foster accessibility and affordability of fruits and vegetables for promoting these among African American women.

The constructs of emotional transformation and practice for change are unique to MTM and have not been reified by previous interventions. The SAVOR intervention demonstrated the utility of emotional transformation in influencing fruits and vegetables consumption among African American women. The process of psychodrama was utilized to demonstrate the usefulness of this construct during a SAVOR session; utilization of this process is recommended by future interventions. Likewise, the SAVOR intervention also found the construct of practice for change to be a beneficial tool supporting an effective behavior change to promoting an increase in consuming more fruits and vegetables. This construct entails “active reflection” and “reflective action” on the desired behavior change. In the SAVOR intervention, this was manifested through social media and app/journaling. Future interventions can utilize these approaches in fostering this construct.

The construct of changes in social environment has been effectively used in the *Steps for a New You* intervention and *Healthy Eating and Harambee* intervention.^13,14^ SAVOR intervention also lends credence to use of changes in the social environment or provision of social support as an effective means to foster behavior change with regard to fruits and vegetables consumption among African American women.

The results of the process evaluation of the SAVOR intervention show that this intervention is both feasible and is highly acceptable among African American women. Understandably, the satisfaction was higher among women in the SAVOR group when compared to the traditional knowledge-based intervention because of the interactive approach utilized by the SAVOR intervention. The program also showed high degree of fidelity to the way it was planned to indicate its replicability in future efforts.

SAVOR utilized the faith-based settings for recruitment of African American women. These settings seem to be conducive to recruiting participants and future interventions aiming to change this behavior in this group can make use of these settings. Early involvement of the pastors and permission from the congregation is vital for such efforts.

### Limitations of the study

The present study had some limitations. First, the data were collected through self-reports which have the potential for several biases such as acquiescence bias, recall bias, dishonesty, exaggeration, etc. which may skew the results. However, this is the only method for collecting data about attitudes or the constructs of MTM. For recording the behavior observations could have been used but were not feasible due to constraints of resources. Future studies can utilize observations. Second, while the sample size was enough for an ORBIT IIb trial, a larger sample could enhance the power and ability to conduct subgroup analyses. Third, an efficacy trial has the potential to sometimes overestimate the intervention’s effect size when implemented for practice in a clinical setting, which was a limitation.^40^ Finally, a per-protocol analysis method for data analysis was used as opposed to intention-to-treat analysis which could introduce bias due to compromised randomization. However, the attrition in the trial was very small so the effect would have been minimal and is justified for pragmatic trials.^39^

### Implications for practice and future research

The primary benefit of MTM is that it has extracted salient constructs from all the contemporary theories to craft precision interventions that can be delivered in a relatively short period of time as was the case with SAVOR intervention. Based on this evaluation it can be said that SAVOR is an efficacious intervention for promoting fruit and vegetable consumption among African American women. This SAVOR intervention adds to the list of evidence-based interventions for promoting fruits and vegetables consumption among African American women. The logical next steps for the SAVOR intervention should be to test it in Phase III efficacy trials and Phase IV effectiveness trials of the ORBIT model.^25^ For effectiveness studies, the SAVOR intervention would need to be implemented at a larger scale in real-world settings with training of trainers who can deliver the intervention at multiple sites. A training manual that facilitates such training can be made available. Greater use of technology in further refinement of the SAVOR intervention should also be a goal for future research in augmenting this intervention. There is need to extend this model to beyond just African American women to other racial groups and to both genders. The study drew a sample from faith-based African American churches. There is a need to mobilize community members from other avenues as well such as patient care settings, community centers, recreation facilities, and other such settings.

## Conclusion

The study developed and tested a fourth-generation intervention based on MTM to promote fruit and vegetable consumption among African American women. This was called as SAVOR. The SAVOR intervention was found to be efficacious and established the strength of MTM in designing precision interventions. Almost all the constructs of MTM and self-reported consumption of fruits and vegetables improved in the SAVOR program when compared to a knowledge-based program over an eight-week period. SAVOR can be replicated for future efficacy and effectiveness trials.

## Ethical approval

The study was approved by the Institutional Review Board (IRB) (Protocol #0128-18). The participation was voluntary. All study participants provided informed consent for participation and could withdraw at any time from the study of any reason. Participants in both study arms were given an incentive of $10 and each participating church was given a $100 donation.

## Competing interests

None.

## Funding

The primary investigator donated personal funds to support this study.

## Authors’ contributions

MS and LB conceptualized the study. This research was implemented solely by LB. MS, LB and HS analyzed the data. The first draft was prepared by LB and MS with subsequent reviews and revisions completed by all authors. All authors reviewed the final draft and gave approval to publish. All authors agreed to be accountable for all aspects of the work in ensuring that questions related to the accuracy or integrity of any part of the work were appropriately investigated and resolved.

## Disclaimer

The study is based on the dissertation of the first author.

## Acknowledgments

We wish to thank the pastors, participants and churches in this study: Tinnin Road Church of Christ, Solid Rock International Ministries, New Dimensions International Church and Grace Inspirations Church. Gratitude is also extended to The Mississippi Urban League and Jackson State University’s School of Public Health.

**Table 1:** Summary of the demographic variable’s distribution: Comparison of experimental (n=27) and comparison groups at pretest (n=28)

**Variable**	**Subgroups**	**Experimental** **No. (%)**	**Comparison** **No. (%)**	**Chi-square value**	***P*** **value**
Education	Did not finish	2 (7)	2 (7)	6.55	0.36
	High school degree	6 (22)	13 (48)		
	Associate degree	5 (18)	6 (22)				
	Bachelor’s degree	5 (18)	2 (7)				
	Masters	7 (25)	3 (11)				
	Professional degree	1 (3)	1 (3)				
	Doctorate degree	1 (3)	0 (0)				
	Total	27 (100)	27 (100)				
Employment	Student	1 (4)	5 (19)	10.63	0.06
	Employed for wages	13 (50)	10 (38)		
	Self-employed	2 (8)	2 (8)		
	Unemployed	0	3 (12)		
	Homemaker	0	2 (8)		
	Retired	10 (38)	4 (15)		
	Total	26 (100)	26 (100)		
Income	Less than $10 000	3 (11)	7 (25)	8.45	0.13
	$10 000-$19 999	4 (14)	7 (25)		
	$20 000-$29 999	4 (14)	4 (14)		
	$30 000-$39 999	3 (11)	5 (19)		
	$40 000-$49 999	3 (11)	2 (7)		
	$50 000	10 (37)	2 (7)		
	Total	27 (100)	27 (100)		
Live in a or house, apt mobile home	Owned by you or someone in this household with a loan or mortgage	15 (55)	11 (41)	4.8	0.18
	Owned by you or someone in this household free and clear	9 (33)	7 (26)		
	Rented for cash rent	3 (12)	10 (33)		
	Total	27 (100)	28 (100)		
Marital Status	Now Married	11 (44)	10 (35)	8.45	0.13
	Widowed	6 (24)	3 (11)		
	Divorced	3 (12)	5 (18)		
	Separated	0	2 (7)		
	Never Married	5 (20)	8 (29)		
	Total	25 (100)	28 (100)		
Age	Mean (St. Dev)	53.74 (13.94)	47.93 (19.97)		0.22

**Table 2:** Comparison of means and standard deviations of subscales scores in experimental (n=27) and comparison groups (n=28) at pre-test

**Variable**	**Possible Range**	**Experimental Group** **Mean (SD)**	**Comparison Group** **Mean (SD)**	***P*** **value**
Participatory dialogue		7.24 (6.02)	2.43 (5.0)	0.003
Advantages	-20.00–20.00	15.52 (4.26)	11.14 (4.34)	0.001
Disadvantages	0.00–20.00	8.28 (3.62)	8.71 (3.86)	0.635
Behavioral confidence	0.00–20.00	10.52 (4.77)	8.57 (4.39)	0.18
Change in physical environment	0.00–12.00	6.26 (2.91)	5.21 (3.05)	0.27
Intention to initiate fruit and vegetable consumption	0.00– 4.00	2.21 (0.88)	1.43 (0.88)	0.19
Emotional transformation	0.00–12.00	6.27 (2.9)	4.53 (2.98)	0.03
Practice for change	0.00–12.00	5.76 (3.09)	4.60 (2.71)	0.25
Change in social environment	0.00–12.00	6.4 (3.10)	4.29 (2.92)	0.03
Intention to Sustain fruit and vegetable consumption	0.00–4.00	1.8 (1.07)	1.61 (1.19)	0.84
Fruit consumption		1.15 (0.68)	1.46 (0.96)	0.12
Vegetable consumption		1.63 (0.98)	1.43 (0.69)	0.37
Fruit and vegetable consumption		2.74 (1.03)	2.89 (1.45)	0.66

**Table 3:** Comparison of means and standard deviations of subscale scores in experimental and comparison groups (pre-test/post-test/follow-up)

**Variable**		**Experimental**			**Comparison**				
	**Possible Range**	**Pre-test** **Mean (SD)**	**Post-test Mean (SD)**	**Follow up** **Mean (SD)**	**Pre-test** **Mean (SD)**	**Post-test** **Mean (SD)**	**Follow up** **Mean (SD)**	**Group x time** ***P*** **value**	**Partial** **eta-squared**
Constructs:									
Participatory dialogue		7.24 (6.02)	9.84 (6.16)	9.76 (6.30)	2.42 (5.0)	1.39 (6.77)	2.75 (3.64)	0.142	--
Advantages	0.00–20.00	15.52 (4.26)	16.68 (3.62)	18.12 (2.50)	11.14 (4.34)	11.25 (5.36)	12.85 (2.94)	0.680	--
Disadvantages	0.00–20.00	8.28 (3.62)	6.88 (3.86)	8.36 (5.24)	8.71 (3.86)	9.85 (4.82)	10.10 (2.33)	0.151	--
Behavioral confidence	0.00–20.00	10.52 (4.77)	14.28 (4.10)	16.64 (2.98)	8.57 (4.39)	9.85 (4.46)	9.35 (2.16)	0.000	0.176
Changes in physical environment	0.00–12.00	6.26 (2.91)	9.03 (2.77)	9.92 (1.80)	5.21 (3.05)	6.03 (2.87)	5.10 (1.89)	0.000	0.212
Intent to initiate FVC	0.00–4.00	2.21 (0.88)	3.34 (0.70)	3.84 (0.38)	1.43 (0.88)	2.07 (0.98)	1.71 (0.94)	0.000	0.176
Emotional transformation	0.00–12.00	6.27 (2.95)	9.23 (2.43)	10.84 (1.61)	4.53 (2.98)	6.25 (3.39)	4.18 (2.03)	0.000	0.258
Practices for change	0.00–12.00	5.76 (3.09)	8.96 (2.54)	9.96 (1.85)	4.60 (2.71)	5.35 (2.93)	3.71 (2.15)	0.000	0.262
Changes in social environment	0.00–12.00	6.4 (3.10)	9.40 (2.40)	10.24 (1.8)	4.29 (2.92)	5.95 (3.32)	4.92 (2.5)	0.000	0.176
Intent to sustain FVC	0.00–4.00	1.8 (1.0)	3.24 (0.88)	3.8 (0.50)	1.61 (1.19)	2.07 (1.01)	1.75 (0.84)	0.000	0.314
Fruit consumption		1.15 (0.68)	2.35 (0.84)	2.12 (0.81)	1.46 (0.96)	1.39 (1.19)	1.29 (0.89)	0.000	0.174
Vegetable consumption		1.63 (0.98)	2.42 (1.17)	2.92 (0.89)	1.43 (0.69)	1.93 (0.94)	1.96 (0.69)	0.020	0.072
FVC		2.77 (1.03)	4.77 (1.34)	5.04 (1.46)	2.89 (1.45)	3.32 (1.82)	3.25 (1.4)	0.000	0.193
